# Superspreading Wetting of Nanofluid Droplet Laden with Highly Dispersed Nanoparticles

**DOI:** 10.1021/acs.langmuir.4c03347

**Published:** 2024-12-02

**Authors:** Eita Shoji, Akira Hoshino, Tetsushi Biwa, Masaki Kubo, Takao Tsukada, Takaaki Tomai, Tadafumi Adschiri

**Affiliations:** † Department of Mechanical Systems Engineering, Tohoku University, Sendai, Miyagi 980-8579, Japan; ‡ Department of Chemical Engineering, Tohoku University Sendai, Sendai, Miyagi 980-8579, Japan; § New Industry Creation Hatchery Center, Tohoku University Sendai, Sendai, Miyagi 980-8579, Japan; ∥ Frontier Research Institute for Interdisciplinary Sciences, Tohoku University Sendai, Sendai, Miyagi 980-8578, Japan; ⊥ WPI-Advanced Institute for Materials Research (WPI-AIMR), Tohoku University Sendai, Sendai, Miyagi 980-8577, Japan

## Abstract

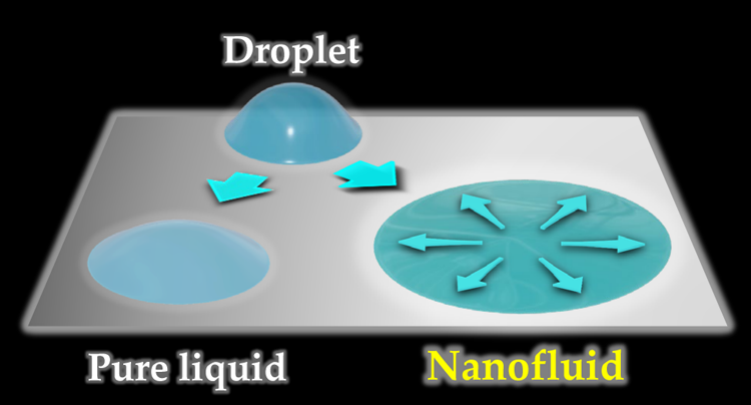

Wetting of nanofluids containing highly dispersed nanoparticles of single-nanometer size was investigated, as these nanoparticles can persist within a nanometer-scale liquid film near contact line, potentially causing significant changes in wetting characteristics. We discerned distinctive superspreading wetting, featured by temporal indices (0.29 to 0.46) in the relationship between contact radius and time. We employed a phase-shifting imaging ellipsometer to measure droplet shape, including the nanometer-scale liquid film and nanoparticle layer after drying. The liquid film shapes differed from pure liquids at micrometer-scale but not at nanometer-scale. Furthermore, surface tension measurements and substrate surface energy control contributed to unraveling these characteristics. These findings differentiated the observed superspreading wetting from the mechanisms proposed in existing studies of aqueous surfactant solutions.

## Introduction

Superspreading wetting is a phenomenon where a droplet on a solid substrate exhibits enhanced spreading dynamics, resulting in faster and larger spreading than that of a pure droplet. The initial observation of superspreading wetting dates back to 1964, in a system where a surfactant-laden water droplet was deposited onto a hydrophobic substrate.^1^ Subsequently, dozens of studies have explored diverse surfactant molecular structures and substrate species, with several comprehensive review articles published.^2−4^ Superspreading wetting is typically analyzed using the exponent of time in the relationship *R* ∝ *t*^α^, where *R* and *t* are the contact radius and time, respectively. Following Tanner’s law,^5^ the exponent α becomes 0.1 and the exponent exceeds 0.1 in the superspreading wetting. Although a definitive threshold value remains undefined, reported values of α have ranged between 0.25 and 1.^6−10^

It is crucial to emphasize that the explanation for superspreading wetting transcends the mere alteration in surface tension resulting from surfactant addition. Rather, mesoscale forces arising from the distribution and structure of the surfactants within the droplets underlie this phenomenon. While earlier studies attributed the driving force to the Marangoni effect induced by the surface tension gradients between the apex and contact line of the droplet,^8^ recent experimental findings have refuted the involvement of the Marangoni effect.^11^ Molecular dynamics simulations have indicated the formation of a surfactant bilayer in a liquid film near the contact line.^12^ A recent study also maintained that surfactants adsorb at the gas–liquid and solid–liquid interfaces, causing the liquid film near the contact line to exhibit a rolling behavior.^3^ Despite considerable research efforts, the underlying mechanism driving superspreading wetting in aqueous surfactant solutions, discovered a significant period ago, remains an ongoing challenge. Given that superspreading wetting in aqueous surfactant solutions remains a discovery without a fully elucidated mechanism, it is meaningful to explore the possibility of finding this phenomenon in other systems.

In this paper, we present findings on superspreading wetting with α = 0.46 using highly dispersed nanofluids, without the use of surfactants for nanoparticle dispersion, i.e., without the surfactant-induced superspreading wetting mechanism described above. To observe superspreading wetting, nanoparticles with surfaces modified with organic materials were used. These nanoparticles showed high dispersibility in organic solvents, even without the use of surfactants for dispersion. Additionally, the liquid used was an organic solvent with lower surface tension than that of water, making it challenging for nanoparticles to adsorb at the gas–liquid interface. Consequently, the superspreading wetting reported here deviates from previously reported mechanisms. The main objective of this study is to highlight the differences from existing superspreading wetting phenomena and describe the unique attributes inherent in the nanofluid-wetting process.

## Experimental Section

### Materials

Surface-modified nanoparticles, synthesized via a supercritical hydrothermal method,^13,14^ were used in this study. The surfaces of the core CeO_2_ nanoparticles were modified with decanoic acid to render them highly dispersible in organic solvents. Transmission electron microscopy (TEM) revealed an average primary particle size of 6.0 nm, as shown in Figure S1(a,b). *n*-alkanes (Fujifilm Wako Pure Chemical) were used to discern variations arising from systematic changes in the properties. The nanoparticles were mixed and centrifuged to prepare nanofluids with concentrations of up to 5 wt %. Dynamic light scattering (DLS) measurements confirmed that the average size of nanoparticles in all nanofluids ranged from 5 to 6 nm, as depicted in Figure S1(c). This is consistent with TEM measurements, affirming nanoparticle dispersion.

Si wafers were selected as substrates due to their smooth surface and suitability for measuring the nanoliquid film near the contact line. In all experiments, new, unused substrates were cleaned with piranha solution and then used, i.e., all substrates were not reused after the wetting experiment. This precaution was taken because the nanoparticles could remain on the substrate even after cleaning. To investigate the effect of solid substrate surface energy, silanized substrates modified by octadecyltriethoxysilane (ODS, Combi-Blocks) were prepared, in addition to substrates subjected solely to cleaning following the use of piranha solution. The surface energies of the cleaning-only and silane-treated substrates were 62.7 and 30.9 mJ/m^2^, respectively, using the Owens–Wendt method,^15^ as presented in Figure S2 and Table S1. The surface roughness of the untreated and silane-treated substrates was also measured using scanning probe microscopy (SPM), yielding average surface roughness values of 0.46 and 0.088 nm, respectively. Comprehensive details on the sample preparation and evaluation processes are provided in the Supporting Information.

### Methods

**Figure 1:**
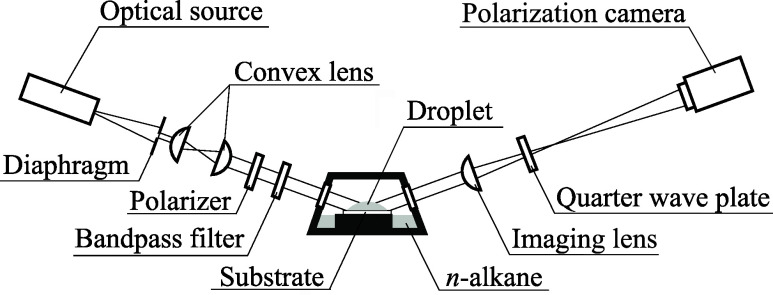
Schematic of experimental apparatus equipped with phase-shifting imaging ellipsometer capable of measuring thickness of liquid films on nanometer-micrometer scale. Sealed container with a liquid tank that suppresses evaporation of droplets was set at the test section of ellipsometer.

The experimental setup is illustrated in Figure 1. A phase-shifting imaging ellipsometer^16,17^ was employed to measure the shape of the entire droplet, including the liquid film extending from the apparent contact line, and to unveil the features of the observed superspreading wetting. The phase-shifting imaging ellipsometry is a measurement technique that incorporates the phase-shifting technique of optical interferometry into ellipsometry. Ellipsometry extracts information about a sample’s optical properties and thickness by analyzing the polarization ellipticity of reflected light when obliquely incident polarized light, consisting of p- and s-polarized components, interacts with the sample. In this method, the ellipticity of the measured light is determined by assessing the phase difference Δ and the amplitude ratio Ψ between the p- and s-polarized components of the measured light. A phase-shifting technique is employed to measure Δ and Ψ for each pixel in two dimensions from multiple images captured by the camera. When the sample comprises three phases (substrate, target thin film, and surrounding material), and the complex refractive index of each phase is known, the thickness of the target thin film can be determined analytically from Δ and Ψ.^18^ This analysis facilitates the measurement of nanometer-scale thickness for each pixel, specifically capturing the shape of a liquid film with a thickness of approximately 100 nm or less, suitable for measuring thin liquid films such as step-like liquid films due to structural disjoining pressure^19^ and precursor films.^20−22^ For liquid films exceeding 100 nm thick, optical interferometry was employed based on the measured phase difference Δ.^17^

Previous studies by the authors employed a three-step algorithm for phase-shifting imaging ellipsometry,^16,17^ though its temporal resolution was somewhat limited by two factors: the need for synchronized operation between the polarizer and camera, and the requirement for polarizer rotation. In contrast, polarization cameras with individual polarizers for each pixel have recently become available, enabling the acquisition of polarization information across multiple directions within a single image. Since many polarizing cameras feature imaging pixels with polarizers oriented in four distinct directions, a four-step algorithm is proposed herein. While the error sensitivity may differ between the three-step and four-step algorithms, there is no fundamental difference in the measured film thickness Consequently, a new phase-shifting imaging ellipsometer was developed and employed in this study.

An overview is provided here based on modifications in the optical setup compared to our previous studies. We employed a light-emitting diode (LED, SOLIS-623C, Thorlabs) with a central wavelength of 623 nm as the light source. Due to the low coherence of the LED, a diaphragm (CP20S, Thorlabs) and two lenses (N-BK7 plano-convex lenses with an antireflection coating, Thorlabs) were initially used to produce collimated light with enhanced coherence. Subsequently, the emitted light passed through a Glan-Thompson prism (10GT04AR.14, Newport) to achieve a polarization state of π/4, ensuring equal intensity of the p- and s-polarized components incident on the sample. Because of the broad wavelength distribution of the LED, a bandpass filter (FLH632.8–3, Thorlabs) selectively transmitted light at 632.8 nm. This optical configuration was designed to optimize the light quality necessary for ellipsometry, mitigating adverse effects such as the speckle patterns observed when laser light is employed in imaging ellipsometry. The incident angle on the sample was set to 70°, and the light reflected from the sample surface, after passing through the imaging lens and quarter-wave plate (10RP04–24, Newport), was captured using a polarization camera (VCXU-50MP, Baumer).

In the phase-shifting technique utilized, the intensities at azimuth angles of the polarizer (0, π/4, π/2, and 3π/4) were captured using a polarization camera, and the following equations were employed to measure Δ and Ψ.
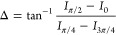
1
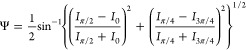
2where *I*_0_, *I*_π/4_, *I*_π/2_, and *I*_3π/4_ represent the intensities at the azimuth angles of the polarizers, positioned in each pixel of the polarization camera, corresponding to 0, π/4, π/2, and 3π/4, respectively. By deriving Δ and Ψ, ellipsometric analysis was conducted to determine the nanometer thickness for each pixel, particularly to capture the shape of the liquid film with a thickness of less than approximately 100 nm, a value that may vary slightly depending on the specific target sample in this study. For liquid films thicker than 100 nm, optical interferometry was utilized based on the acquired Δ information.

To extract the film thickness from Δ and Ψ using ellipsometric analysis with a three–phase model^18^, which includes the substrate, target thin film, and surrounding material, where nanofluids are treated as a single phase without distinguishing between the liquid and nanoparticles, it is crucial that the nanoparticles do not exhibit characteristic interactions at the optical source wavelength. Therefore, the absorption spectra of the nanofluids were examined using an ultraviolet–visible (UV–vis) spectrometer and it was confirmed that there is no characteristic peak at 632.8 nm, as shown in Figure S1(d). Consequently, an effective medium approximation (EMA)^23^ was employed for the ellipsometric measurements. The refractive index of the nanoparticles was estimated to be 1.85 based on the volume fractions of the surface modifier and core particles (the complex refractive index of CeO_2_ is 2.33–i0^24^). Given the dispersion of nanoparticles in the liquids at the concentrations investigated in this study, the refractive indices of the nanofluids deviated by less than 1% compared to those of pure organic solvents. Consequently, the refractive indices of the liquids listed in Table 1, were used to measure the liquid films. Regarding the measurements of the thickness distributions of the nanoparticle layers after drying, the EMA is also applicable to modeling surface roughness layers. Here the ellipsometric analysis was conducted under the assumption that the liquid had evaporated, treating the target thin film as a single phase composed of a mixture of air and nanoparticles at a specific volume fraction. There are some theoretical limitations,^25^ none of which apply to the present samples. Furthermore, as it is challenging to determine the volume fraction of solids in measurements involving surface roughness, the volume fraction is generally assumed to be 0.5. Therefore, the refractive index of 1.42, which was also determined here under the same assumption of 0.5, was used to measure the thickness distribution of the nanoparticle layers.

**Table 1:** Complex Refractive Indices at 20°C^26,27^

material	complex refractive index *n*–i*k*
*n*-hexane	1.376–i0
*n*-heptane	1.387–i0
*n*-octane	1.397–i0
*n*-nonane	1.404–i0
*n*-decane	1.415–i0
Si	3.883–i0.020
Air	1.000–i0

To mitigate organic solvent evaporation due to its high vapor pressure and intradroplet convection, such as capillary flow,^28,29^ Marangoni convection,^30−32^ and contact line instability associated with evaporation,^33^ a sealed container with an organic solvent bath was positioned in the test section of the ellipsometer. This container maintained a saturated condition, effectively suppressing solvent evaporation. It was necessary to wait at least 60 min after closing the container to ensure that the air inside was saturated, as shorter waiting times could cause a small amount of evaporation in this experimental setup. Under these conditions, a nanofluid droplet with a size of 0.1 ± 0.05 μL was deposited onto the substrate using a microsyringe (7105KH PST-3, Hamilton).

Isothermal conditions were maintained throughout the experiment due to the potential for two thermal effects. The first arises when the substrate and droplet temperatures surpass the ambient temperature, resulting in slight droplet evaporation. This can induce intradroplet convections and lead to droplet disappearance. The second effect is the growth of an adsorbed film, which occurs when the ambient temperature within the closed container exceeds that of the substrate and droplet. It was observed that the adsorbed films grew to several tens of nanometers even at slightly reduced substrate temperatures. To mitigate these effects and establish isothermal conditions, the closed container was constructed from copper and designed with a high thermal capacity to minimize temperature fluctuations during the experiment. The room temperature was regulated, and the temperatures of the closed container and substrate were monitored using platinum resistance thermometers (R003 1YRP631, CHINO).

## Results and Discussion

**Figure 2:**
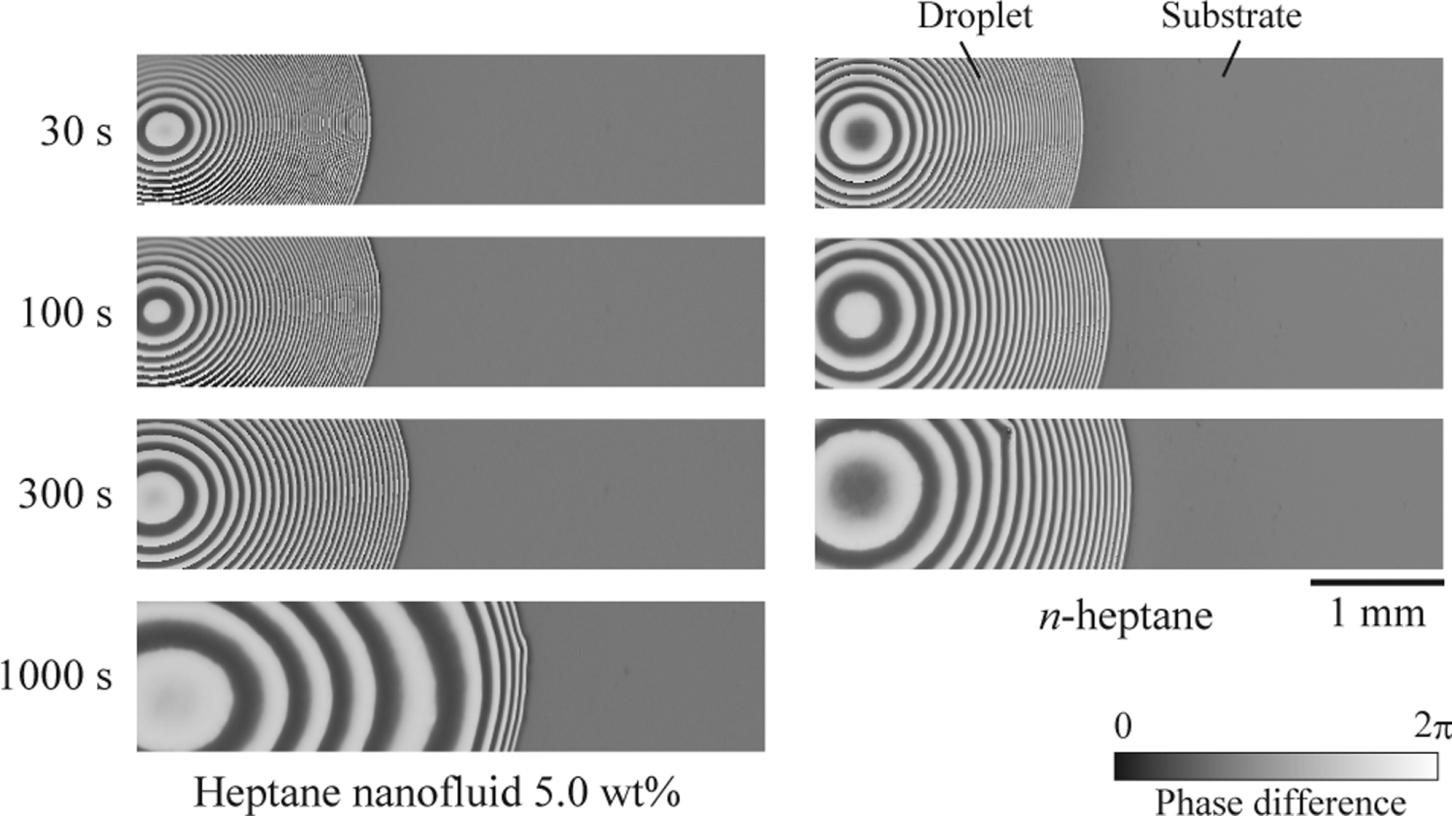
Visualization images near contact line of *n*-heptane droplet and 5 wt % nanofluid droplet composed of *n*-heptane and decanoic acid-modified CeO_2_ nanoparticles deposited on Si substrate. Gray scale color denotes phase difference between p- and s-polarization states in ellipsometry and represents contour line of the liquid film thickness.

Figure 2 presents images of droplets deposited on substrates in a specific area near the contact line. Each image illustrates the distribution of the phase difference Δ between the p- and s-polarization states in the ellipsometry, indicating the contours of the liquid film thickness. The samples depicted are *n*-heptane and a nanofluid composed of *n*-heptane and decanoic acid-modified CeO_2_ nanoparticles. These droplets were deposited on Si substrates cleaned without the silane coupling treatment. In this visualization, an area of interference fringes with a micro liquid film coexists with a uniformly colored region representing the substrate. The segment from the substrate to the first interference fringe constitutes the nanoliquid film area. From the visualization results, it is evident that the droplets exhibit axisymmetric spreading. The *n*-heptane droplet spread over the substrate, and the contact line halted after approximately 300 s. The nanofluid initially spread similarly to the pure solvent. However, after 300 s, the central region of the droplet began to flatten, resulting in a contact radius significantly larger than that of the pure solvent, eventually exceeding twice its size. In other words, superspreading wetting of the nanofluid was observed.

**Figure 3:**
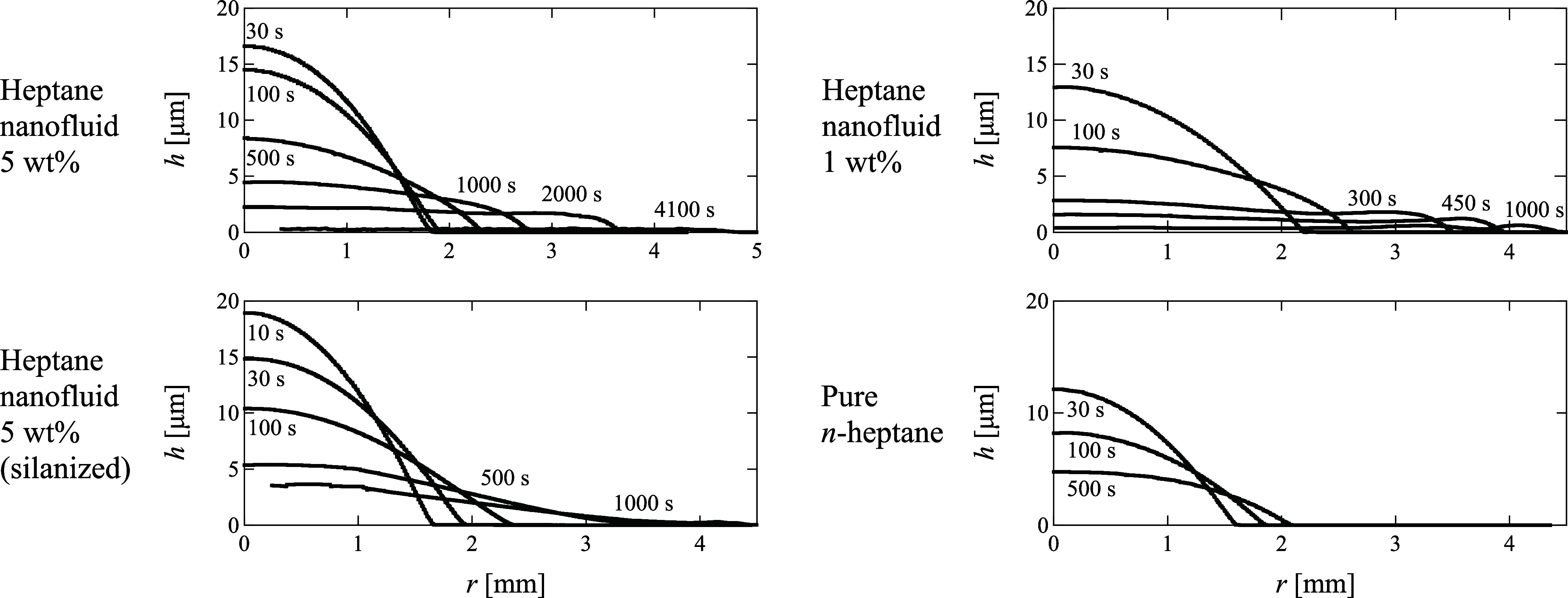
Micrometer-thick liquid film shapes of droplets in radial direction *r* from droplet center. All samples have *n*-heptane as organic solvent. Nanoparticle concentrations are 0, 1, and 5 wt %, respectively, and substrates are either clean or silanized substrates.

For a quantitative evaluation, the temporal evolution of the liquid film thickness *h* at the micrometer scale for all nanofluids with *n*-heptane is illustrated in Figure 3. All the droplets initially maintained their spherical shape. Subsequently, the nanofluid droplets flattened in the central region, exhibiting a characteristic pancake shape. This droplet shape resembles that reported for bicomponent droplets with strong Marangoni convection.^34^ This trend persisted as the nanofluid droplets continued to spread.

Considering previous studies indicating that the surface energy of the substrate correlates with the exponent α in the relationship *R* ∝ *t*^α^ for aqueous surfactant solutions^35^ and that nanoparticle adsorption at the solid–liquid interface alters the wettability of nanofluids,^36^ we modified the surface energy of the substrate through silane coupling treatment. According to force modeling between surface-modified nanoparticles and the substrate,^37^ the silane coupling treatment reduces the adhesion forces of the nanoparticles to the substrate. Moreover, it is worth noting that the electrostatic forces are negligible because the charge on the surface of the nanoparticles is zero in organic solvents.^38^ As depicted in Figure 3, the nanofluid droplet on the silane-treated substrate did not exhibit the pancake shape observed in the other two nanofluids, although it deviated from a spherical shape and showed greater extension compared to *n*-heptane.

**Figure 4:**
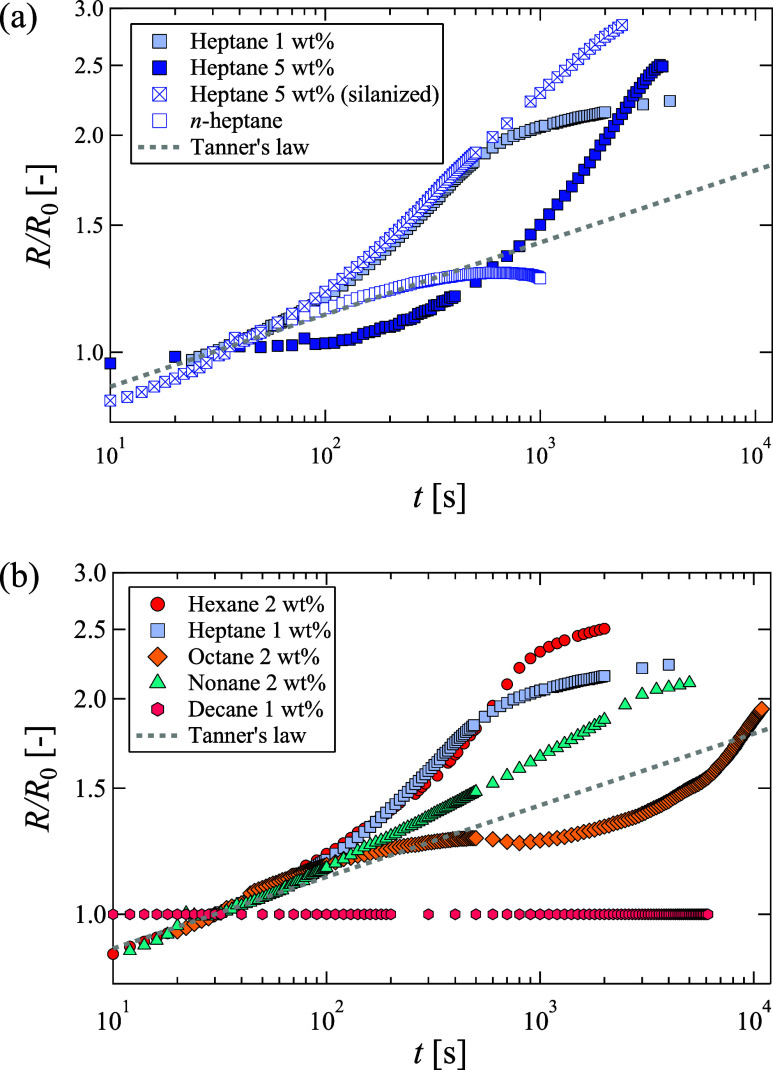
Relationship between dimensionless contact radius *R*/*R*_0_ and time *t* for nanofluids. (a) Variations in nanoparticle concentration and substrate surface energy using *n*-heptane as organic solvent. (b) Effect of individual organic solvents on clean substrate.

To quantitatively characterize the observed superspreading wetting with the exponent α, Figure 4 presents a log-scale plot of the dimensionless contact radius *R*/*R*_0_ against time *t* for the droplets, with the contact radius normalized by the value at 30 s after deposition *R*_0_. The dashed line corresponds to Tanner’s law, i.e., *R* ∝ *t*^0.1^. An important factor in the discussion of superspreading wetting is the exponent α in the relationship *R* ∝ *t*^α^. Therefore, Figure 4 presents the experimental data from a single experiment for each condition, as the relationship can vary slightly due to minor differences in droplet volume or evaporation. As will be discussed later, the heptane nanofluid with the highest exponent α produced values of 0.45, 0.45, 0.48, and 0.45 across four experiments, confirming the reproducibility. Here, the contact line is identified as the location of a 25 nm thick film, because the conventional definition based on inflection points near the contact line^17^ is inapplicable to this phenomenon. This limitation arises from the fact that the droplets demonstrating superspreading wetting do not maintain the spherical shape characteristics of pure liquids, as shown in Figure 3. Furthermore, several 1000 s after the droplet was deposited, it became very thin, and the liquid film extended beyond the first interference fringe (approximately 150 nm). A liquid film thickness of 25 nm was below the interference fringes but exceeded the thickness of the adsorption film, enabling a more precise identification of the contact radius. This measurement was performed with high precision using the phase-shifting imaging ellipsometer.

The relationships between *R*/*R*_0_ and *t* for different nanoparticle concentrations and substrate surface energies in heptane nanofluids are illustrated in Figure 4(a). The contact radius of the *n*-heptane droplet adhered to Tanner’s law, *R* ∝ *t*^0.1^, until 100 s, after which it gradually decreased owing to its small droplet volume and slight evaporation. Spreading obeying Tanner’s law was observed for other *n*-alkanes without nanoparticles. In contrast, while these nanofluids initially followed Tanner’s law, they progressively increased their exponent α with time. The nanofluids exhibited larger exponents, reaching a maximum of α = 0.46, exceeding 0.1. This is close to the range observed in previous studies on surfactant-laden waters.^6−10^ Although superspreading wetting of the nanofluid was observed under the three different conditions, they exhibited different trends. The two solutions with higher concentrations continued to spread for more than 1000 s, while the 1 wt % nanofluid ceased spreading at approximately 1000 s. Because superspreading wetting was observed on both substrates with different surface energies, we consider it unlikely that the mechanism of nanoparticle adsorption to the substrate and the lowering of the coefficient of friction were responsible. Furthermore, this phenomenon differs from that of existing surfactant solutions, in that it begins to exhibit superspreading wetting after a certain period.

**Figure 5:**
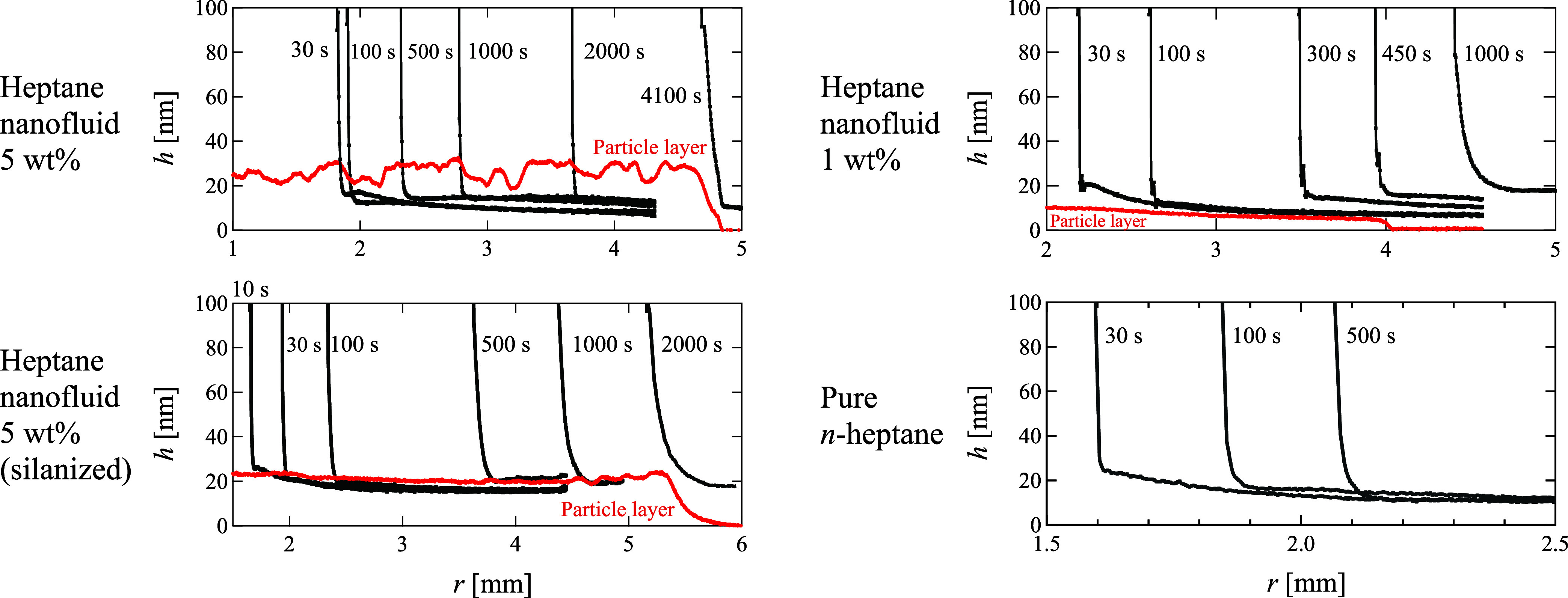
Nanometer-scale liquid film profiles of droplets along radial direction *r*. All samples correspond to those depicted in Figure 3, wherein *n*-heptane serves as organic solvent, nanoparticle concentrations range from 0 to 5 wt %, and substrates include both clean and modified ones. The thickness distribution of the nanoparticle layer subsequent to solvent evaporation, as measured by unsealing and evaporating the liquid 5000 s postdroplet deposition, is also shown as red lines.

Figure 5 illustrates the temporal evolution of the liquid film thickness *h* at the nanometer scale for all the nanofluids with *n*-heptane. At the nanometer scale, the radial thickness did not reach 0 nm because of the presence of an adsorbed film, which was a consequence of the saturation condition.^39,40^ The thickness of the adsorbed film exhibits significant variation with vapor pressure near the saturation vapor pressure and temperature. However, the isothermal conditions established in the aforementioned experimental setup mitigated temporal variations in the film thickness.

The liquid film presented a smooth downward convex curve resembling the liquid film shape observed in precursor films,^17,20,22^ a phenomenon not unique to nanofluids. In addition, the formation of a flat liquid film resulting from bilayer formation, as demonstrated in molecular dynamics simulations of surfactant solutions,^12^ was not observed. The thickness distributions of the nanoparticle layers after solvent evaporation are shown in Figure 5, measured 250 s after releasing the closed condition at 5000 s postdeposition. First, under all conditions, the nanoparticle layer did not exhibit characteristic patterns caused by liquid evaporation, such as coffee ring, and had a relatively uniform thickness distribution. In addition to forming a thicker nanoparticle film, the nanoparticles in the 5 wt % nanofluid remained in the region near the contact line for a longer time, and the nanoparticle layer in the 1 wt % nanofluid matched the contact line position at 450 s. Figure 4 shows that the spreading of the 1 wt % nanofluid slowed from 500 to 1000 s, whereas the 5 wt % nanofluid continued to spread during the experiments. Figure 5 demonstrates the agreement between the tip position of the particle layer at *r* = 4.0 mm and the contact radius at 450 s. It can be deduced that nanoparticles must be present in the liquid film near the contact line to induce superspreading wetting.

The thickness gradient of the nanoliquid film on the silanized substrate is more gradual compared to that on the unsilanized substrate. The roughness of the nanoparticle layer after evaporation is also reduced, likely due to weaker adsorption forces on the substrate,^37^ the behavior of nanoparticles within the nanoliquid film region differs, leading to potential changes in the nanoliquid film shape. However, the occurrence of superspreading wetting on both substrates, despite their differing surface energies, indicates that this phenomenon is unlikely to be driven by a mechanism involving reduced friction caused by nanoparticle adsorption onto the substrate.

### Comparative Analysis with Proposed Mechanisms in Previous Studies

Numerous studies on nanofluid wetting have explored the effects of alterations in surface tension and viscosity resulting from the addition of nanoparticles.^41^ However, little attention has been paid to superspreading wetting. It is imperative to ascertain whether the observed superspreading wetting is attributable to these changes in the thermophysical properties. Given the crucial role of the solute present at the gas–liquid interface in the proposed mechanisms of superspreading wetting in aqueous surfactant solutions, we investigated changes in the wetting phenomenon by varying *n*-alkanes with distinct surface tensions, as illustrated in Figure 4(b). While these nanofluids, except for the decane nanofluid, initially adhered to Tanner’s law, the hexane and heptane nanofluids progressively increased their exponents α. Conversely, the α of octane nanofluid initially decreases and then increases after a certain time. Although α of the nonane nanofluid is larger than that of pure solvents, it is smaller than that of the aforementioned nanofluids. The decane nanofluids did not expand immediately after deposition. Therefore, the nonane nanofluid appeared to be situated at the boundary of the solvent, inducing superspreading wetting.

To confirm whether the phenomenon resulted from a change in the surface tension, we also measured the surface tension of each sample using the pendant droplet method.^42^ The surface tension of 5 wt % heptane nanofluids measured was 19.7 ± 0.3 mN/m, coinciding with the literature surface tension of *n*-heptane at 19.8 ± 0.1 mN/m, indicating that the nanoparticles do not alter the surface tension and do not adsorb at the gas–liquid interface. In contrast, the surface tension of the decane nanofluid that did not spread at all was 22.2 ± 0.2 mN/m, slightly lower than that of *n*-decane (22.7 ± 0.4 mN/m). Although the difference is small and requires careful consideration to determine whether nanoparticles are indeed adsorbed at the gas–liquid interface, these results are contrary to observations in aqueous surfactant solutions, suggesting that the formation of Marangoni convection and bilayers is unlikely.

From a rheological perspective, the viscosity of nanofluids remains nearly constant within the studied concentration range, showing shear-thickening behavior under high concentration conditions.^43^ This suggests that a reduction in viscosity is unlikely to drive the observed spreading. Instead, the persistence of superspreading wetting increased with higher concentrations, as depicted in Figure 4(a). It was also noted that slight evaporation occurs in this system. Evaporation of droplets on the substrate is typically more pronounced near the contact line than at the center of the droplet. Consequently, nanoparticles localized in the thin liquid film near the contact line potentially had a locally high concentration. Moreover, a notable characteristic of this phenomenon is the onset of superspreading wetting after a certain elapsed time, particularly after the droplets thinned, as shown in Figure 3. It is also noteworthy that nanoparticles were present in the nanoliquid film near the contact line, which played a role in inducing this phenomenon.

One of the distinctive driving forces of nanofluids is the structural disjoining pressure, which originates from the arrangement of nanoparticles in a liquid film near the contact line.^19^ This pressure, analogous to the solvation forces, induces the formation of steps in the liquid film shape near the contact line, characterized by a thickness approximately equal to the diameter of the nanoparticles. Numerical fluid dynamics simulations that consider this force for nanofluid droplets on a substrate also demonstrate the formation of step-like liquid films.^44,45^ Based on these simulations, we can infer that the step height and width are of the order of tens of nanometers and several hundred micrometers, respectively, which can be measured by phase-shifting imaging ellipsometry. However, step-like liquid film shapes were not observed in Figure 5. Furthermore, the nanoparticle concentrations in the nanofluids exhibiting structural disjoining pressure were more than 10 times larger than those in the present study, which is another feature distinguishing them from previously reported instances of observed superspreading wetting.

## Conclusions

In summary, we investigated the wetting behavior of nanofluids containing highly dispersed nanoparticles. Specifically, we experimentally measured the time variation of the droplet contact radius, droplet shape ranging from nanometer to micrometer scales, and the nanoparticle layer after droplet drying using a phase-shifting imaging ellipsometer. We observed superspreading wetting in nanofluid droplets containing decanoic acid-modified CeO_2_ nanoparticles dispersed in *n*-hexane, *n*-heptane, and *n*-octane, with index values ranging from 0.29 to 0.46 for the time exponent α in the relationship *R* ∝ *t*^α^. In contrast, nonane nanofluids almost followed Tanner’s law α = 0.1, and the decane nanofluid exhibited no spreading α = 0.

Superspreading wetting was therefore observed in the three nanofluids with lower surface tension and viscosity. In other words, superspreading wetting occurred in nanofluids where nanoparticles were less likely to migrate to the gas–liquid interface, and surface tension measurements confirmed that the nanoparticles did not alter the surface tension. This indicates that the mechanism is distinct from that of aqueous surfactant solutions,^3,8,11,12^ which are the primary focus of superspreading wetting studies. Furthermore, phase-shifting imaging ellipsometry measurements of droplet shape revealed that during superspreading wetting, droplets deviated from a spherical shape in the micrometer scale, while no specific shape was observed in the nanometer scale, corresponding to the size scale of the nanoparticles. This finding means that the superspreading wetting observed in this study does not originate from the development of structural disjoining pressure in the nanofluids,^19,44,45^ nor from the structuring of surfactant solutions near the contact line.^3,12^ Moreover, the viscosity of the nanofluids remains constant within the concentration range examined in this study and exhibits shear-thickening behavior at higher concentrations,^43^ making it unlikely that changes in spreading are driven by reduced viscosity. Therefore, the observed superspreading wetting, as revealed by these measurements, could not be explained by the mechanisms proposed in previous studies.

This unobserved phenomenon in nanofluids is expected to increase in importance with the development of novel nanoparticles and nanofluids. While we have delineated the features of this phenomenon, it is also crucial to evaluate each interface for surface-modified nanoparticles, such as those used in this study. To this end, we are analyzing the interfacial parameters of complex interfaces using molecular dynamics simulations.^46−48^ Based on these fundamental findings, it is anticipated that the driving force behind it will be elucidated in the future, enabling control over the wetting phenomena.

## Supporting Information Available

Nanofluids preparation procedures; transmission electron microscopy images of nanoparticles; DLS and UV–vis results of nanofluids; substrate cleaning procedures; surface energy measurement of substrates (PDF)
