# Supplementary material for: Superspreading Wetting of Nanofluid Droplet Laden with Highly Dispersed Nanoparticles
Source: Langmuir. 2024 Dec 2;40(50):26509–16. doi: 10.1021/acs.langmuir.4c03347 (PMC11656740; doi:10.1021/acs.langmuir.4c03347)
Supplement: Supplementary file 1 — la4c03347_si_001.pdf [file la4c03347_si_001.pdf]

Supporting Information: superspreading wetting of nanofluid droplet laden with highly dispersed nanoparticles

Eita Shoji,[*,†] Akira Hoshino,[†] Tetsushi Biwa,[†] Masaki Kubo,[‡] Takao Tsukada,[¶] Takaaki Tomai,[§] and Tadafumi Adschiri[∥]

†*Department of Mechanical Systems Engineering, Tohoku University Sendai, Miyagi, 980-8579, Japan*

‡*Department of Chemical Engineering, Tohoku University Sendai, Miyagi, 980-8579, Japan*

¶*New Industry Creation Hatchery Center, Tohoku University Sendai, Miyagi, 980-8579, Japan*

§*Frontier Research Institute for Interdisciplinary Sciences, Tohoku University Sendai, Miyagi, 980-8578, Japan*

∥*WPI–Advanced Institute for Materials Research (WPI-AIMR), Tohoku University Sendai, Miyagi, 980-8577, Japan*

E-mail: eita.shoji@tohoku.ac.jp

Contents

1 Nanofluids **S-2**

2 Substrates **S-2**

References **S-5**

1 Nanofluids

Decanoic acid-modified CeO_2 nanoparticles were synthesized using a supercritical hydrothermal method.[S1,S2] Following the washing and selection steps described in Ref,[S2] the synthesized nanoparticles were lyophilized and stored. Subsequently, the lyophilized nanoparticles were mixed with the organic solvents centrifuged to remove the aggregated nanoparticles. Figure S1 presents transmission electron microscopy (TEM) images of nanoparticles, the particle size distribution of nanoparticles derived from the TEM images, the particle size distribution of nanoparticles in nanofluids using the dynamic light scattering (DLS) method, and the absorption spectrum of the nanofluid measured by an ultraviolet–visible (UV-Vis) spectrometer. The TEM revealed an average primary particle size of 6.0 nm. DLS measurements confirmed that the average size of nanoparticles in the nanofluid ranged from 5 to 6 nm, consistent with TEM measurements, thereby affirming nanoparticle dispersion. To further apply the three-phase model to ellipsometry, as described in the main text, the absence of a characteristic interaction of the nanoparticles at the source wavelength was investigated by measuring the absorption spectra of the nanofluids. It can be seen that there is no characteristic peak at 633 nm.

2 Substrates

The Si substrate underwent a thorough cleaning process before experimentation. Initially, it was immersed in ethanol and subjected to ultrasonic irradiation for 10 min with deionized water using an ultrasonic cleaner. After drying, the substrates underwent a washing step with piranha solution, prepared by combining H_2SO_4 and H_2O_2 at a volume ratio of approximately 7:3, at 60 °C for 6 h. Subsequently, the Si substrate was removed from the piranha solution and underwent ultrasonic irradiation with deionized water. Silanization of the Si substrates was performed to explore their effects on surface energy. Following the piranha cleaning method described earlier, the Si substrate was immersed in a cyclohexane solution containing

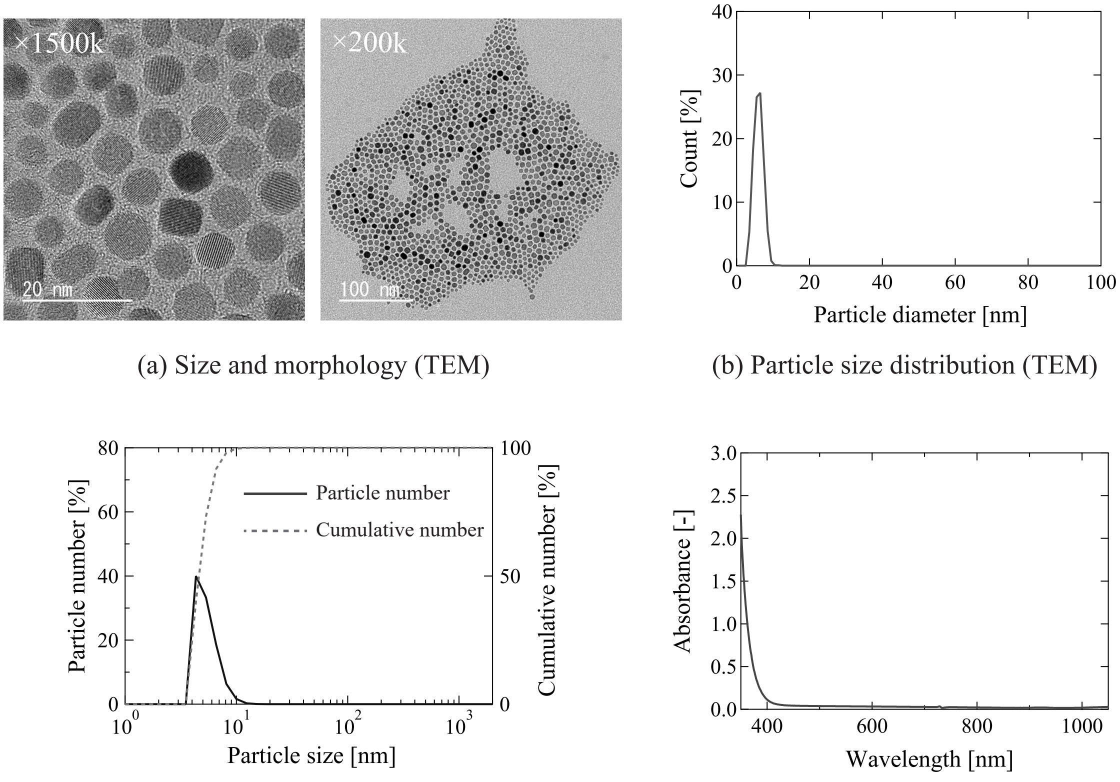

(a) Size and morphology (TEM)

(b) Particle size distribution (TEM)

(c) Particle size distribution (DLS)

(d) Absorption spectrum (UV-Vis)

Figure S1: (a) Size and morphology of nanoparticles observed by TEM and (b) particle size distribution based on observation results. (c) Particle size distribution (volume fraction) of nanoparticles in heptane nanofluid at 5 wt% measured by DLS. (d) Absorption spectrum of decanoic acid-modified CeO_2 nanoparticles in organic solvent (n-heptane) measured by UV-Vis spectroscopy. From (a) to (c) it was confirmed that the nanoparticles exhibited a unimodal size distribution and were well-dispersed in the organic solvent. Furthermore, since no characteristic spectrum is observed at 633 nm, which is the wavelength used in the phase-shifting imaging ellipsometer, the effective medium approximation is applicable.

0.01 M octadecyltriethoxysilane (ODS, Combi-Blocks) at 60°C for 1 hour. It was then ultrasonically irradiated in acetone for 10 minutes and then in deionized water for 10 minutes. Finally, the Si substrates were heated at 120°C for 2 h to evaporate the liquid on the substrate and subsequently stored in a desiccator under reduced pressure.

To confirm whether the surface properties of the untreated and silanized substrates had changed, the surface energy of the substrate was measured based on the Owens-Wendt method $\sigma_{\mathrm{L}}(1+\cos\theta_{\mathrm{e}}) = 2\sqrt{\sigma_{\mathrm{L}}^{\mathrm{p}}\sigma_{\mathrm{S}}^{\mathrm{p}}} + 2\sqrt{\sigma_{\mathrm{L}}^{\mathrm{d}}\sigma_{\mathrm{S}}^{\mathrm{d}}}$, where σ_{L} $(= \sigma_{\mathrm{L}}^{\mathrm{d}} + \sigma_{\mathrm{L}}^{\mathrm{p}})$ is the surface tension of the liquid and σ_{S} is the surface energy of the substrate.[S3] Superscripts p and d denote

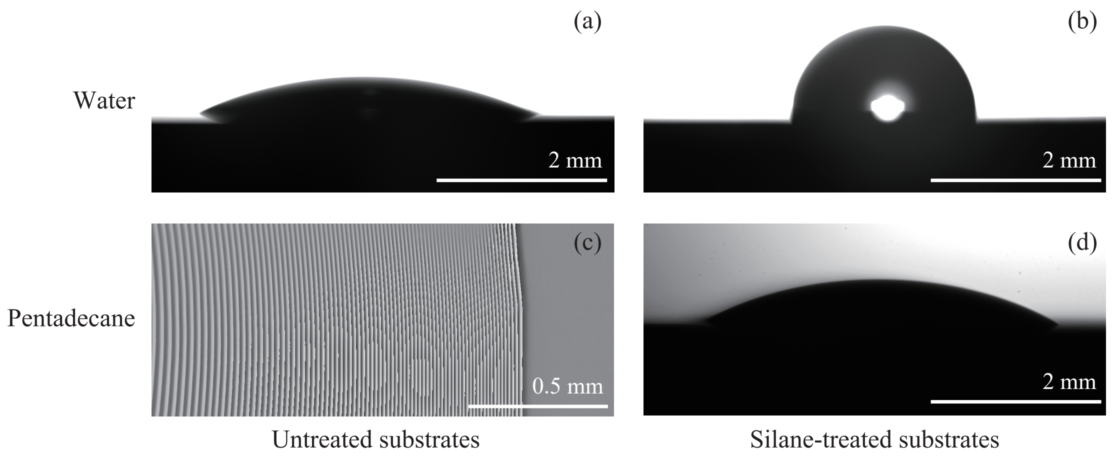

Figure S2: Contact angle measurement of droplets on the substrates for surface energy determination of the substrates by the Owens-Wendt method. Each figure shows (a) deionized water on the untreated substrate, (b) deionized water on the silane-treated substrate, (c) pentadecane on the untreated substrate, and (d) pentadecane on the silane-treated substrate. In (a), (b), and (d), the droplets are observed from the side. Conversely, (c) is obtained using the phase-shifting imaging ellipsometer.

the polar and dispersive components of surface energy, respectively, and θ_e denotes the equilibrium contact angle. Using the measured contact angles θ_e of two liquids of known surface energy, here water ($\sigma_\mathrm{L}^\mathrm{p} = 51.0$ mN/m, $\sigma_\mathrm{L}^\mathrm{d} = 21.8$ mN/m[S3]) and pentadecane ($\sigma_\mathrm{L}^\mathrm{p} = 0$ mN/m, $\sigma_\mathrm{L}^\mathrm{d} = 27.1$ mN/m[S4]), the two components of the surface energy of the substrate, $\sigma_\mathrm{S}^\mathrm{d}$ and $\sigma_\mathrm{S}^\mathrm{p}$, were determined. Figure S2 shows the visualization images of the contact angle measurements, and the contact angles of the droplets to the substrates were determined. The measured surface energies are summarized in Table S1.

Table S1: Surface energy of the substrates used.

Substrate	$\sigma_\mathrm{S}^\mathrm{d}$ [mN/m]	$\sigma_\mathrm{S}^\mathrm{p}$ [mN/m]	σ_S [mN/m]
Untreated	27.1	35.6	62.7
Silane-treated	24.1	6.73	30.9

References

(S1) Zhang, J.; Ohara, S.; Umetsu, M.; Naka, T.; Hatakeyama, Y.; Adschiri, T. Colloidal ceria nanocrystals: A tailor-made crystal morphology in dupercritical water. *Adv. Mater.* **2007**, *19*, 203–206.

(S2) Tomai, T.; Tajima, N.; Kimura, M.; Yoko, A.; Seong, G.; Adschiri, T. Solvent accommodation effect on dispersibility of metal oxide nanoparticle with chemisorbed organic shell. *J. Colloid Interface Sci.* **2021**, *587*, 574–580.

(S3) Owens, D. K.; Wendt, R. C. Estimation of the surface free energy of polymers. *J. Appl. Polym. Sci.* **1969**, *13*, 1741–1747.

(S4) Jasper, J. J. The Surface Tension of Pure Liquid Compounds. *J. Phys. Chem. Ref. Data* **1972**, *1*, 841–1010.
